# Exploring physician leadership perceptions: Insights from first- and final-year medical students

**DOI:** 10.1371/journal.pone.0314082

**Published:** 2024-11-21

**Authors:** Sari Huikko-Tarvainen, Timo Tuovinen, Petri Kulmala

**Affiliations:** 1 Faculty of Medicine, University of Oulu, Oulu, Finland; 2 Research Unit of Health Sciences and Technology, University of Oulu, Oulu, Finland; 3 Medical Research Center, Oulu University Hospital, Oulu, Finland; The University of York, UNITED KINGDOM OF GREAT BRITAIN AND NORTHERN IRELAND

## Abstract

**Background:**

Leadership competence is increasingly recognized as a critical priority for all physicians, but new graduates often feel only partially prepared for leadership roles. However, integrating more leadership education into the already saturated medical curriculum poses challenges regarding timing and implementation. This study explores this issue by comparing perceptions of medical students during their academic journey to determine if leadership education could begin at the onset of medical studies.

**Methods:**

In 2021, internet-based questionnaires were administered to first- and final-year medical students at the University of Oulu, Finland. Participation rates were 100% (116/116) for first-year students and 98% (107/109) for final-year students. Responses to the open-ended question, "How should physicians be led?" were analyzed using qualitative inductive content analysis with thematization.

**Results:**

The study identified three main thematic categories common to both groups: (1) traits, (2) leadership approach, and (3) healthcare culture. The theme of leadership approach was further divided into two subcategories: science-oriented leadership based on practice approach and goal-oriented leadership with support as needed. The theme of traits was divided into seven subcategories: education, role model, communication, empowerment, ethics, autonomy, and collegiality. No notable differences emerged between the two groups.

**Conclusions:**

Consistent perceptions about physician leadership throughout medical education suggest that leadership education could commence from the first year. It can thus be integrated throughout the existing longitudinal learning of the medical curriculum. Integration into the existing curriculum could facilitate the development of leadership skills without extending the curriculum’s content.

## Introduction

Involving physicians in the leadership process of healthcare significantly impacts performance, quality, and safety of care. These impacts can be measured by various process and outcome indicators [1–3]. Consequently, leadership competence is expected to become an essential skill for all physicians in both medical education and healthcare settings in the future [4].

Healthcare is undergoing rapid changes, making it crucial for physicians to develop leadership skills to effectively guide these transformations. The importance of leadership development at every hierarchical level in the medical profession is recognized by medical regulators worldwide [5–10]. Additionally, integrating applied interdisciplinary leadership education into curricula for medical and other health professions could strengthen students’ confidence in their ability to effect change in healthcare and assume future leadership roles [11].

While not all physicians will become formal leaders in health organizations, most will serve as informal leaders within their communities or practices [12]. According to the Finnish Medical Association’s recent study of physicians (n = 4 885) conducted every fifth year, 20% of working age respondents’ (under the age of 65) work includes formal front-line leadership work. The majority of the respondents considered leadership education for physicians insufficient. Even though 28% of all respondents were quite interested or very interested in formal leadership positions, interest has decreased since 2018, when 34% of physicians were very interested or quite interested in formal leadership positions [13]. Thus, introducing leadership skills to all undergraduate medical students early and throughout their education could be beneficial. These skills are no different from other skills taught to medical students. This approach also provides a solid foundation for continuing professional leadership education after medical school and pursuing formal physician leadership positions as career choices [12].

Moreover, the COVID-19 crisis heightened the need for leadership and management competencies. This situation has required physician leaders and physicians at all levels in the healthcare system to act as managers—establishing processes and structures—and as leaders capable of creating new visions and inspiring action [14]. In daily healthcare practice, physicians at all levels are expected to exhibit various leadership skills as part of their daily responsibilities. Additionally, since medical students in Finland are permitted to temporarily work as doctors in formal doctor vacancies under certain circumstances, this partially applies to them as well [15].

Previous studies have posited that physicians need both management and leadership skills [16]. However, a recent systematic review revealed that medical students and new graduates struggle to define leadership, even though they understand its role in current practice, and recognize the importance of leadership and management, despite their acknowledged significance [17]. This confusion may stem from a lack of leadership education in formal medical curricula, an insufficient understanding of theoretical leadership models, or the belief that leadership skills are innate. Additionally, there are concerns that gender biases may influence perceptions of leadership, potentially complicating future workplace dynamics [18]. Furthermore, it has been argued that formal leadership education probably does not lead to changes in leadership understanding but can refine these perceptions. Thus, there is a call for workplace-based learning that integrates theoretical knowledge with practical insights into workplace contexts, relationships, and organizational structures [19].

New graduates often view leadership as individualistic and hierarchical and feel only partially prepared for leadership roles. This preparedness is linked to increasing responsibility, experience, and tenure. Furthermore, the leadership communication skills of senior colleagues are rarely questioned [17]. Hence, medical students typically have a traditional view of leadership, seeing followers as adherents to the leader’s vision and goals [17, 19]. Similarly, second-year resident physicians in hospitals often distinguish sharply between management and leadership, deeming the former inferior [20]. According to a recent Canadian study of medical students, a character, a competence, and a commitment were appreciated in physician leadership; thus, more possibilities to participate in leadership work as well as reflection exercises were suggested for inclusion in leadership education [21], which aligns with the perspectives of more experienced physicians and physician leaders [22, 23].

All in all, while physicians’ leadership competence is crucial [4], the current undergraduate medical education does not provide sufficient leadership training [16, 24]. The challenge lies in integrating more leadership education into an already dense medical curriculum. Thus, research-based knowledge is needed to understand when and how to incorporate leadership studies into the medical curriculum effectively. However, studies in this context are scarce. One approach to expand our understanding is to investigate the stability of medical students’ perceptions on leadership during medical school. Therefore, we have begun addressing this issue by comparing the perceptions of the first-year and the final (sixth)-year medical students on how physicians should be led. To our knowledge, no such previous studies exist.

Since perceptions can influence actions, motivation, and behavior [3]—both presently and in the future—it is crucial to research and value these perceptions. Addressing this need and fostering a comprehensive understanding of physician leadership across all stages of a medical profession including medical students as physicians and physician leaders of tomorrow, is both timely and relevant. Hence, we believe that the findings of this study can be universally beneficial in planning leadership education for medical students.

### The context of the study

In Finland, undergraduate medical education (Licentiate of Medicine, 360 ECTS credits) spans six years [25]. Medical students who have completed their first four years of study are temporarily allowed to perform physician activities under supervision in specialized medical care units or primary health center wards but not in other roles within health centers [15].

In 2021, the Finnish Association of Junior Doctors (FAJD) advocated for reforming leadership and management studies in medical education to align better with healthcare service needs. They emphasized the importance of individual awareness, progressing from personal management to organizational and systemic leadership. The FAJD proposed unified leadership studies across universities, with resources allocated for education and practical application [24]. Since 2009, postgraduate Finnish medical education has included compulsory first-line leadership training [26]. In 2020, common competence goals highlighted leadership skills for graduating physicians, reflecting the significance of understanding leadership roles in healthcare [27]. However, studies suggest a lack of coherent structure in leadership studies within the Finnish medical curriculum [28]. While moderately covered in basic education [28], specialization studies vary between universities and often focus on irrelevant topics [29]. Urgent corrective actions are necessary to address healthcare’s demanding leadership requirements [4] and the impending leadership gap due to retirements [30].

In sum, an adequate leadership education is not consistently offered [16], but the content of medical curricula is already crowded. Therefore, we need research-based knowledge to understand how we can organize leadership studies without extending the curriculum. Medical students’ perceptions are previously scarcely heard in this issue even though they are physicians and physician leaders of tomorrow. Especially considering the possible changes in mindset over the generations, it is essential to research and value also medical students’ perceptions, as perceptions may influence actions, motivation, and behavior [3]. Hence, this study could be pivotal in determining the timing and implementation of leadership education universally.

## Materials and methods

### Study design

The survey instrument was meticulously developed through an extensive review of existing literature on physician leadership, including the perceptions of physicians and medical students regarding physician leadership and leadership education within medical curricula. Utilizing a survey methodology was considered suitable for this study due to its effectiveness in efficiently collecting data from large samples within a limited timeframe [31]. Additionally, since this study was conducted during the COVID-19 pandemic, extensive face-to-face meetings were generally discouraged. Before full implementation, the survey underwent a pretesting phase among the authors to assess the clarity of survey items and identify potential ambiguities or challenges in interpretation. The authors’ research team, consisting of professionals in medicine, medical education, and physician leadership research, brought significant expertise from academic, clinical, and physician leadership research environments. The feedback gathered during this pretesting phase was aimed to ensure the questionnaire’s relevance to the study population, as well as its clarity and comprehensibility [31–34].

We distributed the survey to all 116 first-year and 109 final-year medical students at the University of Oulu, Finland, in 2021. Respondents were contacted via email with an invitation letter to participate in the survey. The letter was sent to the first-year medical students prior to the initial introduction lecture of the " Introduction to medical profession "course. This compulsory first-year course (4 ECTS) is designed to orient new medical students to the medical curriculum, study techniques, cultural aspects of studying medicine, and the medical profession. It covers topics such as professionalism, the various roles of a medical doctor, pathways to becoming a doctor, and the importance of collegiality. Additionally, it emphasizes time management and introduces students to health services. By the course’s conclusion, students gain a comprehensive understanding of medical study at the university, develop essential time management abilities, and grasp the diverse roles of medical professionals in practice. They appreciate the significance of professionalism and collegiality both in their studies and future careers. Moreover, students acquire knowledge of medical ethics, comprehend the structure and function of healthcare systems, and recognize the legal frameworks governing healthcare organization. Furthermore, students become familiar with the professionals working in healthcare centers, appreciate the interdisciplinary nature of medical work, and understand the imperative of teamwork in patient care. They recognize the importance of interactive skills within the medical profession, identify personal strengths and areas for development in interpersonal interactions, and acknowledge the potential for skill enhancement. Students also gain proficiency in cardiopulmonary resuscitation skills, understanding the factors influencing successful resuscitation, and recognizing situations necessitating immediate intervention. They learn to prevent further injury progression and maintain life support through basic procedures such as cardiac massage and defibrillation. Additionally, they demonstrate the ability to apply treatment recommendations to manage heart failure patients and are motivated to uphold and refine their resuscitation skills through continued practice and training [35].

The same invitation letter was sent to the final-year medical students prior to the initial introduction lecture of the "Leadership in Healthcare" course. This compulsory final-year course (2 ECTS) serves as an introductory exploration of essential themes in leadership and organizational dynamics. It underscores the significance of leadership and management within the scope of a physician’s responsibilities, advocating for a perspective that considers the diverse interests of stakeholders. The overarching aim is to adeptly structure personnel for proficient and innovative performance. Upon completion, students will distinguish the differences between expert and supervisory roles, understand the functionalities of expert organizations, and the fundamentals of good leadership. They will also establish a foundation in economic thinking and its application in clinical decision-making, along with acquiring tools for self-management, leadership development, and honing critical decision-making skills. Participation or non-participation in the survey did not affect passing these courses, as the survey was completely voluntary and not part of the courses’ agenda.

The recruitment period for this study was from September 10th to September 19th, 2021, for first-year medical students and from October 28th to November 4th, 2021, for final-year medical students. The samples of the first- and the final-year students represented the population of interest well as the entire cohorts of the first- and the final-year medical students of the year 2021 were invited to participate in the survey, resulting in high response rates as participation rates were 100% (116/116) for first-year students and 98% (107/109) for final-year students.

### Ethical statement

In the invitation letters sent to the first-year and the final-year medical students, the students were provided with clear instructions outlining the study’s purpose, the voluntary nature of their participation, assurances of confidentiality and anonymity, and their right to withdraw or refuse data use at any point. Consent for data collection was part of the questionnaire and usage in research was obtained from all participants. Data analyses were conducted without personal identification, and no incentives were offered for participation. The study adheres to national and international research ethics standards for non-medical research involving human participants, following the ethical principles outlined by the Finnish National Board on Research Integrity TENK (2019) [36] and the data protection regulations of the European Union. According to Finnish law and ethical guidelines, the study did not require clearance from an ethics committee. Permission for the study was granted by the Faculty of Medicine in accordance with current policies.

### Study questionnaire

The survey was administered electronically using a web-based platform (Webpropol) to ensure convenient access and prompt completion for participants. The final survey instrument included question covering students’ perspectives on physician leadership. Furthermore, additional inquiries encompassed various facets of doctors’ work experiences (in paid employment as a doctor) during medical education, along with questions concerning respondents’ age, gender, and previous educational degrees before entering medical school, but these were not incorporated into the present study. The English translation of the survey, which was originally in Finnish, is available in the S1 Appendix. The open-ended question of the survey analyzed in this study was as follows: “How should physicians be led?" This single qualitative question was chosen to provide us the possibility to gain a wide range of answers that were not tied to specific, narrow issues. The responses were digitally saved and coded for easier analysis. The answers yielded a total of eighteen A4-sized pages (Calibri font, 12 point, single spacing).

### Data analyses

We employed a qualitative approach [37] utilizing inductive content analysis through thematization as our analytical method. This approach aimed to provide a comprehensive description of the studied phenomenon, linking our findings with broader contexts and previous research on the subject [33]. The research was twofold: initially analyzing data from first-year and final-year students separately, followed by comparing the results between the two groups.

Our analysis systematically examined the collected data, focusing on relevant themes and patterns. We identified common or exceptional statements or viewpoints. The process commenced with thorough familiarization with the data, followed by grouping, combining, and organizing elemental codes (words and phrases) into categories reflecting expectations of how physicians should be led. These findings were then further categorized into potential subcategories, which were reviewed and refined until distinct and consistent subcategories emerged.

Subthemes were identified (education, role model, communication, empowerment, ethics, autonomy, collegiality, science-oriented leadership based on a practice approach, and goal-oriented leadership with support as needed) and combined into main themes: (1) traits, (2) leadership approach, and (3) the culture of healthcare. These themes were analyzed both individually and within the overall thematic structure.

Initially, the first author conducted the data-driven analysis twice, after which the data were evaluated by all the authors. Throughout the final analysis and coding phases, the first author consulted with the other authors every 1–2 months. Researcher triangulation was integral throughout this study to enhance and elucidate the research findings [33], with three researchers from different academic backgrounds in the field of medicine investigating the data and cross-checking their interpretations and conclusions. Informative medical students’ (S) citations from the data were selected to illustrate the researchers’ interpretations. Expressions that could compromise anonymity were either removed or altered in the interview excerpts [33]. However, these alterations did not significantly impact the analysis or results, as the modified phrasing was solely considered for use in the excerpts. Finally, the material was ultimately organized into a tabular format.

## Results

The qualitative analysis of our study yielded three main thematic categories common to both first- and final-year medical student groups: (1) traits, (2) a leadership approach, and (3) the culture of healthcare. The theme of a leadership approach was further divided into two subcategories: science-oriented leadership based on a practice approach and goal-oriented leadership with support as needed. Similarly, the traits were divided into seven subcategories: education, role model, communication, empowerment, ethics, autonomy, and collegiality. Upon comparing the results between the two groups, consistent findings emerged (Table 1).

**Table 1:** The results of the first- and the final-year medical students’ perceptions regarding how physicians should be led.

Main themes	Sub themes	The results of the first-year medical students and *citations*	The results of the final-year medical students and *citations*
**Traits**	**Education**	Physician leaders should possess a medical education and be professionals within their respective fields. If the leader does not have a medical background, it is imperative to have a physician with a robust understanding of medical knowledge to serve as a collaborative partner. *A physician is best suited to lead fellow physicians*. *(S93)* *As a professional within their field*. *(S32)*	Successful physician leadership is facilitated by drawing upon the medical experience of a physician leader. Thus, it is crucial for leaders of physicians to possess medical expertise themselves. This entails not only functioning as peers rather than evaluating oneself at a higher level, but also comprehending the realities of clinical work at various stages of a physician’s career, as well as distinguishing between professional and personal life. *Leaders must possess knowledge and understanding of modern physician work*, *including identifying problem areas*, *and understanding the diverse patient population within the field*. *(S42)* *Leading with a support of medical knowledge*. *(S79)* *By functioning as a peer*. *(S39)*
	**Role model**	Physicians should be led with fairness and confidence, setting a good role model from a position of equality rather than hierarchy. Subordinates anticipate being heard, and leadership characterized by equitable treatment and a shared sense of responsibility is anticipated. *With equality*, *respecting and establishing clear frameworks for work and areas of responsibility*. *(S18)* *Above all*, *with understanding and on an equal footing*, *rather than from a hierarchical top-down approach*. *(S33)* *In addition*, *it is always best to lead by a role model*. *(S6)*	Physicians should be led fairly by role model, from the front line, with collegial manners, expertise, and competence. It is essential to listen to physicians and assign responsibilities accordingly. However, a top-down management approach is not sustainable in the long term. *Leading by a role model and on the front line*. *(S13)* *Fairly*. *(S5)* *Top-down management does not work in the long term*. *(S95)* *No micromanagement*. *(S50)*
	**Communication**	A reciprocal communication. A good physician leadership encompasses qualities such as clarity, equality, approachability, and effective management practices aimed at optimizing outcomes. A proficient leader possesses the ability to communicate effectively in all directions. A success in communication translates orders into a success in leadership. The results indicate a preference for collaborative, non-hierarchical communication in leadership. *Engaging in reciprocal discussions is vital; when individuals feel heard*, *it becomes easier to listen*, *thereby shifting the dynamic from issuing orders to effective leadership*. *(S16)* *Excellent leadership embodies clarity*, *equality*, *approachability*, *and adept management*, *ensuring optimal functionality and performance*. *A good leader excels in communicating effectively in all directions*. *(S83)* *From the same level as those being led (S6)*. *Above all*, *with understanding and from the same level*, *not through top-down leadership*. *(S33)*	Effective communication, particularly mutual communication, is imperative for a physician to address challenging issues successfully. This can be facilitated by minimizing hierarchical barriers within the profession. While the leader retains ultimate decision-making authority and responsibility, it is essential to consider subordinates’ perspectives openly and with trust to make decisions that are both credible and inclusive. *By clearly informing*, *openly outlining*, *and establishing common rules and practices with subordinates*. *(S16)* *Engaging in discussions and considering the perspectives of [doctor] peers is essential for effective decision-making*. *However*, *it’s essential that the leader maintains control and direction*, *ultimately guiding the decision-making process towards a conclusive direction*. *(S71)* *By being a peer*, *leading from the same level without elevating oneself*, *and leading humanely through active listening*. *(S39)* *In my opinion*, *listening to employees is important in leadership*. *Top-down leadership does not work in the long run*. *(S95)*
	**Empowerment**	In good physician leadership, efforts are made to identify and leverage each physician’s talents and potentials effectively. Workloads are delegated in a manner that prevents individuals from becoming overwhelmed, ensuring a balanced distribution of responsibilities. *Physicians should be led with the objective of leveraging their individual strengths and finding their fullest potential*. *(S34)* *Delegate tasks in a manner that prevents anyone from becoming overwhelmed*. *(S86)*	Good leadership resembles coaching, aiming to bring out the potential of individuals through support, encouragement, and attentive listening. Valuing openness, honesty, and transparency, a good leader provides constructive feedback in a positive manner, highlighting areas for improvement. They possess a clear vision and guide others towards it, while also addressing problems openly and being willing to collaborate on solutions. *Leading through support*, *encouragement*, *and listening*. *I value a leader who is willing to address grievances openly and demonstrate the commitment to implement necessary changes*. *On the other hand*, *I also appreciate when a leader addresses areas where I can improve in a constructive manner*. *Openness*, *honesty*, *and transparency are qualities I value highly in leadership*. *(S14)* *Demonstrate unequivocal support from a higher authority whom subordinates can rely on for assistance during challenging situations*. *Leadership should be enacted as a form of coaching*, *where the leader objectively identifies areas for improvement in a physician’s work*. *(S15)* *I believe it’s important in leadership to listen to employees and grant them the freedom to organize their work according to their personality*, *strengths*, *and weaknesses…It’s important to prioritize occupational well-being and maintain a positive workplace atmosphere*. *(S95)*
	**Ethics**	A leader’s altruistic behavior is expected in a good physician leadership. *Leader’s primary purpose should not solely revolve around generating personal gain or benefits for themselves or the organization they represent*. *(S76)*	In a good physician leadership, financial gain is not the primary driver. *Not just the glitter of money in the eyes*. *(S28)*
	**Autonomy**	In a good physician leadership, leaders make decisions independently while also engaging in interactive management and leveraging group intelligence. Additionally, they prioritize respecting physicians’ autonomy and stand behind their decisions. *Physicians should be managed with respect for professionalism and autonomy*, *i*.*e*., *interactively*, *listening to the physicians’ point of view*. *(S87)* *Physicians*, *like other professionals*, *require leadership*, *but their professional autonomy must be respected*. *(S24)*	Physicians, as experts in their field, require their autonomy to be respected due to the nature of their work, which involves substantial independent decision-making and responsibility. Consequently, the significance of autonomy in physicians’ roles becomes more apparent when their leader shares a similar background as a physician. Moreover, subordinates greatly appreciate clear instructions for their work and support from higher authorities, especially in challenging situations. As the importance of flexible working conditions continues to be emphasized, successful physician leadership prioritizes being as employee oriented as possible. *Leading with the respect for physician’s autonomy*. *(S11)* *A physician’s role necessitates a lot of independent decision-making and responsibility*. *Because of this*, *physicians need space*, *but clear frames*. *(S72)* *Physicians must be guaranteed autonomy to do their own work*, *but also clear superior support from a higher authority*, *to whom they can turn for help in problematic situations*. *(S15)*
	**Collegiality**	Physicians should be led collegiately, appreciatively, and respectfully with a good cooperation. *With collegiately*, *appreciatively*, *respectfully*. *(S22)* *With collegiately*. *We are all respected professionals and have an extensive education*. *Physicians should not be managed bossily*, *so to speak*, *from above*, *but as colleagues*. *(S81)*	In good physician leadership, collegiality is essential. Therefore, authority should be derived not through coercion or micromanagement, but rather through medical experience. While emphasizing physician autonomy and promoting leadership approaches that are egalitarian, respectful, flexible, and cooperative, it is essential to establish clear and uniform workplace standards applicable to all. This includes responsibilities such as paperwork and emergency shifts, which should be shared among everyone. *Respectfully*, *collegially*. *(S36)* *Equally*, *clear common rules of the game*, *everyone should do unpleasant things (paperwork*, *emergency shifts*, *etc*.*) equally*. *(S16)*
**Leadership approach**	**A science-oriented leadership based on a practice approach**	Physicians should be lead with information based on scientific knowledge and a good situational awareness. A physician leader does not necessarily need to possess the highest level of medical knowledge, although having a deeper understanding of medicine enhances their medical authority. *Based on the evidence-based knowledge*. *I think the most important thing is to lead physicians with information*. *On the other hand*, *it cannot always be assumed that the physician in a leadership position is the "most knowledgeable" or "best" physician*.*…however*, *some kind of authority can also be achieved with deep knowledge*. *I think the leader also needs a kind of social eye for the game*. *(S79)*	Leadership should be grounded in knowledge and effectiveness. However, “a practical eye” is necessary as the consideration of employees’ individual needs—such as family situations, research commitments, and personal health issues—is becoming increasingly important. A generally reasonable leadership approach is suggested, but for physicians, it would likely be most effective if the leader of physicians is also a physician themselves. However, possessing economic knowledge and relevant work experience would be a valuable skill. *Leading with a practical eye*. *Flexibility is an ever-highlighting strength*. *(S20)* *Leading is based on knowledge and effectiveness*. *Flexibility is an asset that is always highlighted*. *The consideration of employees’ individual needs*, *such as family*, *research interests*, *and health factors*, *is steadily growing*. *(S75)* *It probably works best if a physician is the leader of the physicians*. *However*, *e*.*g*., *financial knowledge is essential for a leader*, *so knowledge and work experience of it would not be a bad thing*. *(S60)*
	**A goal-oriented leadership with support as needed**	Physician leadership should have a clear goal, such as improving healthcare operations. Good leadership entails enabling physicians’ work and providing clear instructions for subordinates, facilitating task performance with support is needed. A good leadership fosters the professional development of subordinates and prioritizes their well-being, fostering a people-oriented work environment. *Leadership must have a clear goal*, *for example*, *improving healthcare operations*. *(S85)* *Physicians should be led in a manner that clearly delineates their individual tasks*, *with instructions provided as needed to ensure task performance*. *(S47)* *By providing support in challenging work decisions and prioritizing the professional development of subordinates*, *thereby enabling it*. *(S68)*	To manage a physician leadership successfully, the current issues related to work and work environment should be systematically and clearly informed. While prioritizing a people-oriented leadership approach, it’s also essential to maintain a rational approach to healthcare economics. Demonstrating common sense in behavior is prudent. This entails actively listening to subordinates, openly aligning on common rules and practices, and reaching agreements. Prioritizing occupational well-being is important, and leaders should set a role model in fostering a positive workplace atmosphere and collegiality. Subordinates may require various forms of assistance to carry out their work effectively. However, it’s imperative for leaders to uphold a compassionate attitude in the workplace, valuing humanity in all interactions. Physicians should be actively involved in decision-making processes whenever feasible, given the unique and often stressful nature of their work. *Leading with knowledge*, *determination*, *and collegiality*. *(S100)* *Financial matters should be managed with the assistance of an economist*.*…As an example*, *in primary healthcare*, *various units may incur significantly different expenses due to variations in patient demographics*. *However*, *budget allocations often remain uniform across units because economists may not understand these clinical differences*. *(S20)* *A good physician leadership facilitates the implementation of reasonable scheduling and work organization*, *supports professional development*, *and ensures adequate resourcing to maintain a manageable workload*. *(S44)*
**A culture of healthcare**		In a good physician leadership, there is a comprehensive understanding of all levels of the unique working environment, including its challenges and pain points. *So that you understand the levels of the organization you are leading*, *know all the professions you lead within it*, *and know how to lead well*. *(S62)* *The leader should understand the workload of subordinates and the environment and the work community*. *(S93)* *There is still a significant amount of unnecessary and overlapping work being done in healthcare*, *and I believe that leaders from outside the healthcare sector could offer valuable insights to streamline processes*. *Of course*, *such a manager would require a strong medical perspective in their working*. *partner*. *(S101)*	In physician leadership, it’s imperative for the leader to possess a mastery of both the practical aspects of physicians’ work and operational models, considering their unique features. Additionally, the leader should have a comprehensive understanding of various healthcare domains, encompassing diverse opinions and remaining open-minded to different perspectives. *It would be beneficial for the leader to have experience in practical work and operating models*. *The leader should be a so-called trendsetter*, *someone capable of bringing out the potential of individuals in the most effective manner possible*. *(S96)* *By mastering the subject and various areas within the field*, *by actively listening to multiple opinions and maintaining an open-minded approach*, *supported by medical expertise*. *(S28)*

## Discussion

According to the findings of our study, consistent results were observed across both groups. Given that final-year medical students in Finland are permitted to temporarily work as doctors in formal doctor vacancies under certain circumstances but the first-year students are not [15], we expected the results of the groups to differ from each other. However, the perceptions of both groups closely align with those of experienced physicians and physician leaders reported in the literature [22, 23]. The lack of differences in perceptions between the two medical student groups in our study, as well as in previous literature [22, 23], suggests that leadership may also be influenced by universal principles. Moreover, medical students may share a similar vision of their future professional identity, which could be another confounding factor. Furthermore, identities seem to shape behaviors and experiences [38], which in turn influence identity development within a social context [39]. Additionally, a sense of collective belonging to a professional group [39] may also have impacted the results. Nonetheless, the desire for humane treatment and respect transcends prior experience.

The greater a leader’s medical knowledge is, the more authority he or she commands. Therefore, successful physician leadership is actualized through the leader’s own medical experience and understanding of the realities of clinical work at various career stages. Being a peer and a role model on the front line, while exhibiting collegial manners, expertise, and competence, is vital. If the leader is not a physician, a medically knowledgeable physician as a working partner is necessary. Furthermore, in the light of the results of this study, effective physician leadership is characterized by a science-oriented approach grounded in practical experience. This reflects the need for a leadership approach grounded in broader, evidence-based scientific knowledge, including, for example, economic knowledge, as well as an understanding of physicians’ lives in a broader context, such as family situations, research commitments, and personal health issues. However, leaders are expected to display altruistic behavior, where financial gain is not the primary driver. Hence, understanding all levels of the healthcare environment, with their challenges and pain points, is essential. Additionally, the leadership approach should be goal-oriented, providing clear instructions and support from higher authorities, especially in challenging work situations. Collegiality is essential, and common-sense behavior is wise, while micromanagement or top-down management is ineffective in the long term. Leaders are expected to prioritize occupational well-being and serve as role models in promoting a positive workplace atmosphere and collegiality. These findings align with the previous studies addressing that physicians need both management and leadership skills [16].

Subordinates desire to be heard and engaged in open discussions to align and agree on common rules and practices. Effective communication is key to successful leadership. Mutual communication is necessary to address challenging issues and can be facilitated by minimizing hierarchical barriers. Conversely, effective communication across the profession can help lower these hierarchical barriers. Openness, honesty, and transparency are highly valued, with good leaders providing constructive feedback and proactively addressing problems. Leaders’ decision-making should be independent yet interactive, utilizing group intelligence and respecting physicians’ autonomy, as their work involves significant independent decision-making and responsibility. Furthermore, respecting the autonomy of physicians being led includes listening to their points of view. However, this necessary autonomy does not diminish the importance of effective communication in successful physician leadership. In the light of these findings, medical students appear capable of defining leadership work comprehensively, which differs from but also partially aligns with previous literature. Students understand the leadership role in current practice and recognize the importance of leadership and management [17].

According to previous literature, medical students traditionally view leadership as a relationship in which followers are seen as adherents to the leader’s vision and goals [17, 19]. This perspective contrasts with the findings of our study. Our research identifies a generational shift in attitudes toward this leader-follower relationship compared to previous literature. Instead of favoring top-down leadership, our findings suggest that an approach rooted in mutual respect and valuing egalitarianism and interpersonal connections is more preferred. Based on the results of this study, subordinates want responsibilities, and their professional development should be enabled. Ideal physician leadership resembles coaching, where individuals’ potentials are maximized through support, encouragement, and attentive listening without overburdening anyone. While workplace rules should be uniform, individuals may require different types of support to perform their duties. Despite the growing call for flexible working conditions and a people-oriented, respectful, and cooperative approach, the rational supervision of healthcare economics must not be neglected.

## Conclusions

As new graduates have reported feeling only partially prepared for leadership roles [17], and physicians have expressed that leadership education for physicians is insufficient, [13] there is a clear need to expand leadership education in medical curricula. Understanding medical students’ perceptions of physician leadership is a step toward best identifying practices for leadership education in undergraduate medical education programs.

Our findings reveal no substantial differences in perceptions of physician leadership between first-year medical students and final-year medical students. Furthermore, their perceptions align with those observed in recent studies of Canadian medical students [21], as well as among residents, more experienced physicians, and physician leaders [22, 23]. However, our study identifies a generational shift in the leader-follower relationship compared to previous literature. The medical students in this study do not adhere to the traditional view of leadership, which sees followers as mere adherents to the leader’s vision and goals [17, 19]. Instead, they favor a leadership approach rooted in mutual respect, which suggests a paradigm that values egalitarianism and interpersonal connections over top-down leadership.

Since leadership skills are no different from other skills taught to medical students, introducing leadership training to all undergraduate medical students early and throughout their education can be beneficial [12]. Furthermore, given that the stage of medical education does not appear to influence students’ perceptions of physician leadership, our findings suggest that leadership education need not be postponed; it can commence in the first year and continue throughout the medical education.

The long-term objective is to determine the type of leadership training needed and how it can be effectively incorporated into an already crowded medical curriculum without overburdening students. As the medical curriculum already includes fundamental leadership competencies such as communication and teamwork [40], one possible solution could be to integrate broader leadership education into the existing time-limited longitudinal elements of the medical curriculum [40]. Implemented in this way, it could provide a meaningful context for students to practice and develop leadership skills and leader identity at various stages of their professional development, offer a time- and cost-effective approach [41].

## Strengths, limitations, and future research

This study benefits from sufficiently large sample sizes and excellent response rates. However, several limitations must be considered when interpreting the findings. The study was restricted to first-year and final (sixth)-year medical students from a single university, and many outcomes of leadership interventions are challenging to measure. Additionally, qualitative research inherently has limited generalizability, though similarities may be drawn in comparable research contexts. A survey methodology was employed for its effectiveness in efficiently collecting data from large samples within a limited timeframe [31]. While in-depth interviews could have provided richer interpretations and a more nuanced understanding of the research topic, they also have limitations, such as the potential for individuals to feel marginalized, intimidated, or reluctant to speak openly in individual or group settings [33]. Moreover, the study was conducted during the COVID-19 pandemic, a period when extensive face-to-face interactions were generally discouraged. Future research could examine the evolving perceptions of the same cohort of medical students regarding leadership throughout their medical education and assess whether these perceptions change after completing a leadership course. Additionally, it may be valuable to compare medical students’ perceptions with established theoretical models of leadership.

## Supporting information

S1 Appendix Study questionnaire. (DOCX)

S2 Appendix Human subjects research checklist. (DOCX)
