# Supplementary material for: Exploring physician leadership perceptions: Insights from first- and final-year medical students
Source: PLoS One. 2024 Nov 21;19(11):e0314082. doi: 10.1371/journal.pone.0314082 (PMC11581274; doi:10.1371/journal.pone.0314082)
Supplement: S1 Appendix — (DOCX) [file pone.0314082.s001.docx]

***Study questionnaire***

English translation of the survey, which was originally in Finnish, is available in the Appendix.

All the survey questions in the study were as follows but the open-ended question of the survey analyzed in this study was:” How should physicians be led?"

1. Consent to participate in the study.

- Yes - I give my consent to using the data collected in this questionnaire for scientific research purposes and thus participate in the study.
- No - I do not give permission to use the information from this questionnaire for scientific research purposes.

1. Age.
2. Gender.
3. Highest level of education attained by final year medical students prior to medical school:

- Primary school /Vocational school /High school
- Open ended answers.

1. Have you worked as a doctor (in paid employment as a doctor) during your medical studies?
2. If you have worked as a doctor (in paid employment as a doctor) during your medical studies, how long (in months)? and
3. If you have worked as a doctor (in paid employment as a doctor) during your medical studies, what kind of work (description)?
4. Would you be interested in working as a physician leader in the future?
5. How should physicians be led?
6. How would you describe a good physician leader?
7. Importance of managerial roles based on Mintzberg’s framework in physician leaders’ work was evaluated by using a 5-point Likert scale.
