# Supplementary material for: Exploring physician leadership perceptions: Insights from first- and final-year medical students
Source: PLoS One. 2024 Nov 21;19(11):e0314082. doi: 10.1371/journal.pone.0314082 (PMC11581274; doi:10.1371/journal.pone.0314082)
Supplement: S2 Appendix — (DOCX) [file pone.0314082.s002.docx]

**Human Participants Research Checklist**

***Complete the following if your study involved human participants or human participants’ data. These questions should be addressed for prospective and retrospective studies.***

1. Did you obtain ethics approval for this study?
   - If yes, please upload (file type “Other”) the original approval document you received from your ethics committee. If the original document is in another language, please also provide an English translation.

__X_ Uploaded N/A

- - If you did not obtain ethical approval, please explain why this was not required below.

**Ethical Statement**

In the invitation letters sent to the first-year and the final-year medical students, the students were provided with clear instructions outlining the study's purpose, the voluntary nature of their participation, assurances of confidentiality and anonymity, and their right to withdraw or refuse data use at any point. Consent for data collection was part of the questionnaire and usage in research was obtained from all participants. Data analyses were conducted without personal identification, and no incentives were offered for participation. The study adheres to national and international research ethics standards for non-medical research involving human participants, following the ethical principles outlined by the Finnish National Board on Research Integrity TENK (2019) and the data protection regulations of the European Union. According to Finnish law and ethical guidelines, the study did not require clearance from an ethics committee. Permission for the study was granted by the Faculty of Medicine in accordance with current policies

1. If you prospectively recruited human participants for the study – for example, you conducted a clinical trial, distributed questionnaires, or obtained tissues, data or samples for the purposes of this study, please report in the Methods:
   1. the day, month and year of the **start and end** of the recruitment period for this study.
   2. whether participants provided informed consent, and if so, what type was obtained (for instance, written or verbal, and if verbal, how it was documented and witnessed). If your study included minors, state whether you obtained consent from parents or guardians. If the need for consent was waived by the ethics committee, please include this information.

__X_ Completed ___ N/A

1. If you are reporting a retrospective study of medical records or archived samples, please report in the Methods section:
2. the day, month and year when the data were accessed for research purposes
3. whether authors had access to information that could identify individual participants during or after data collection

___ Completed __X_ N/A
